# Polyamidoamine dendrimer impairs mitochondrial oxidation in brain tissue

**DOI:** 10.1186/1477-3155-11-9

**Published:** 2013-04-04

**Authors:** Gabriella Nyitrai, László Héja, István Jablonkai, Ildikó Pál, Júlia Visy, Julianna Kardos

**Affiliations:** 1 Department of Functional Pharmacology, Institute of Molecular Pharmacology, Research Centre for Natural Sciences, Hungarian Academy of Science, Budapest, Hungary; 2 Department of Biochemical Pharmacology, Laboratory of Chemical Pharmacology, Research Centre for Natural Sciences, Hungarian Academy of Science, Budapest, Hungary

**Keywords:** Nanotoxicity, PAMAM dendrimer, Brain tissue, Calcium enhancement, Mitochondrial depolarization

## Abstract

**Background:**

The potential nanocarrier polyamidoamine (PAMAM) generation 5 (G5-NH_2_) dendrimer has been shown to evoke lasting neuronal depolarization and cell death in a concentration-dependent manner. In this study we explored the early progression of G5-NH_2_ action in brain tissue on neuronal and astroglial cells.

**Results:**

In order to describe early mechanisms of G5-NH_2_ dendrimer action in brain tissue we assessed G5-NH_2_ trafficking, free intracellular Ca^2+^ and mitochondrial membrane potential (Ψ_MITO_) changes in the rat hippocampal slice by microfluorimetry. With the help of fluorescent dye conjugated G5-NH_2_, we observed predominant appearance of the dendrimer in the plasma membrane of pyramidal neurons and glial cells within 30 min. Under this condition, G5-NH_2_ evoked robust intracellular Ca^2+^ enhancements and Ψ_MITO_ depolarization both in pyramidal neurons and astroglial cells. Intracellular Ca^2+^ enhancements clearly preceded Ψ_MITO_ depolarization in astroglial cells. Comparing activation dynamics, neurons and glia showed prevalence of lasting and transient Ψ_MITO_ depolarization, respectively. Transient as opposed to lasting Ψ_MITO_ changes to short-term G5-NH_2_ application suggested better survival of astroglia, as observed in the CA3 *stratum radiatum* area. We also showed that direct effect of G5-NH_2_ on astroglial Ψ_MITO_ was significantly enhanced by neuron-astroglia interaction, subsequent to G5-NH_2_ evoked neuronal activation.

**Conclusion:**

These findings indicate that the interaction of the PAMAM dendrimer with the plasma membrane leads to robust activation of neurons and astroglial cells, leading to mitochondrial depolarization. Distinguishable dynamics of mitochondrial depolarization in neurons and astroglia suggest that the enhanced mitochondrial depolarization followed by impaired oxidative metabolism of neurons may be the primary basis of neurotoxicity.

## Background

Polyamidoamine (PAMAM) dendrimers are hyperbranched “protein-like” polymers with well-defined globular structure and monodispersed, nanoscopic particle size. PAMAM dendrimers have been reported to be able to cross the blood–brain barrier and are used as nanoparticle delivery systems to carry DNA, drugs or imaging agents to the brain [1-3]. Despite its wide application in the brain, only general toxic effects of dendrimers have been studied [4-6], much less information is available about their effects on neural cells.

In our recent study we showed that application of polycationic PAMAM generation 5 (G5-NH_2_) dendrimers induced severe depolarization and subsequent inactivation of hippocampal pyramidal neurons in brain slices. Additionally, cell death after G5-NH_2_ application was also observed in a concentration dependent manner [7]. In the present study we characterize the early intracellular processes sequential to G5-NH_2_ induced neuronal depolarization and explore the effect of dendrimer application on astroglial cells.

In addition to their role in maintaining neuronal function, astroglial cells have been disclosed as active contributors in signal processing [8,9]. Furthermore, we also reported on astroglial signaling independent of neuronal activity in acute brain slices isolated from the rat nucleus accumbens [10]. In general, neurons are more susceptible to oxidative injury than astrocytes, due to their limited antioxidant capacity [11]. During oxidative stress astrocytes support neuronal function by providing antioxidant protection [11,12]. Therefore damage resulting in astrocyte dysfunction leads to increased neuronal death [11]. Oxidative damage in neural tissue can be detected by the loss of mitochondrial membrane potential (Ψ_MITO_), a marker of mitochondrial dysfunction [11,13] that is sensitively coupled to neuronal and astroglial activation and survival [11,14,15]. Ψ_MITO_ is also coupled to intracellular Ca^2+^ regulation [11,13,14,16]. Changes in intracellular Ca^2+^ level indicate activation of neuronal [17,18] or astroglial cells [10,19,20].

Membrane-dendrimer interactions have been studied extensively, supporting the view that cationic dendrimers interact with biological membranes [4,21,22]. Interactions are often followed by cellular internalization of cationic PAMAM dendrimers [23].

Here we report that application of G5-NH_2_ dendrimer induces robust intracellular Ca^2+^ signals in both neuronal and astroglial cells followed by severe Ψ_MITO_ depolarization indicating the disruption of neuronal oxidative metabolism.

## Results and discussion

### Plasma membrane appearance of fluorescently labeled G5-NH_2_ in neuronal and astroglial cells

**Figure 1:**
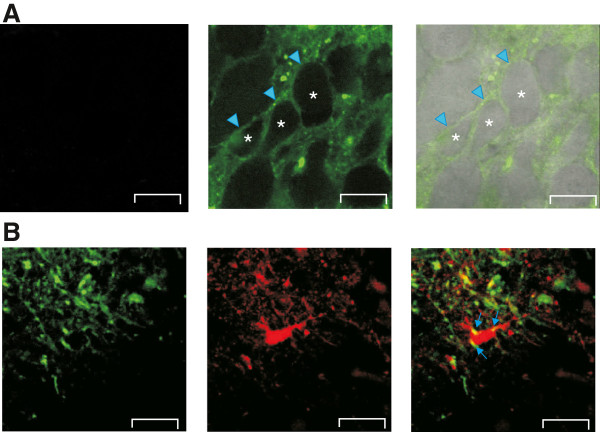
**Plasma membrane appearance of Rhodamine Green conjugated G5-NH_2_ in the rat hippocampal slice after 30 min incubation with 0.1 mg/ml conjugated dendrimer. A**: No fluorescence signal was detected from slices without dendrimer application (*Left*, control) Green fluorescent contour of pyramidal neurons in the hippocampal CA3 area indicates the occurrence of dendrimer mainly in the plasma membrane (asterisks - center of the cells, arrowheads point to the plasma membrane). **B**: Appearance of the green fluorescent dendrimer (*Left*) and the astroglia specific red fluorescent marker SR101 (*Middle*) in the *stratum radiatum* area next to the CA3 pyramidal cells. Yellow spots in the superimposed image (*Right*) indicate localization of the dendrimer to the plasma membrane of the soma and processes of an astroglial cell (arrows). Scale bars: 20 μm.

To determine the localization of G5-NH_2_ in the rat hippocampal slices we covalently conjugated the fluorescent Rhodamine Green dye to G5-NH_2_ and studied the localization of the fluorescently labeled dendrimer with confocal microscopy after 30 minutes of incubation. We found robust plasma membrane appearance of 0.1 mg/ml G5-NH_2_ in the pyramidal layer of acute hippocampal slices (Figure 1A, n = 3 slices). Fluorescent G5-NH_2_ appeared predominantly on the cell membrane of pyramidal neurons, although weak internalization of the dendrimer was also observed (Figure 1A). On astroglial cells a patchy membrane distribution of the green fluorescent G5-NH_2_ was detected as colocalization with the astroglia-specific red fluorescent marker sulforhodamine-101, SR101, Figure 1B, n = 5 slices), suggesting direct interaction between the astroglial cell membrane and the dendrimer (Figure 1B).

### Intracellular Ca^2+^ responses of astroglial cells and neurons

**Figure 2:**
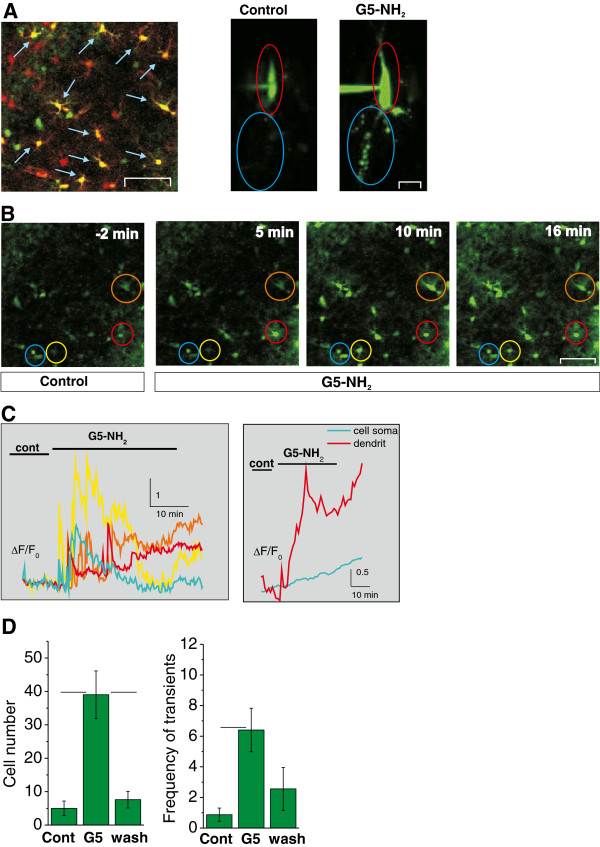
**G5-NH_2_ (0.1 mg/ml, 30 min) induces intracellular Ca^2+^ enhancements in astroglial and neuronal cells as monitored with the fluorescent Ca^2+^ indicator Fluo-4. A**: *Left*: Co-localization (yellow cells pointed by blue arrows) of astroglial cells stained with the astroglia-specific marker SR101 (red) with the Ca^2+^ indicator Fluo-4 (green). Scale bar: 100 μm. *Right*: Representative images of a CA3 pyramidal neuron filled with the membrane impermeable Ca^2+^ indicator MagFluo4 (green) before and after G5-NH_2_ application. Scale bar: 50 μm. Red and blue ovals indicate somatic and dendritic regions of the cell, respectively. **B**: Representative serial images showing Ca^2+^ enhancements in the circled astroglial cells in the *stratum radiatum* of the rat hippocampal slice. Scale bar: 50 μm. **C**: Fluorescence-time plots of the astroglial cells circled in **B** (*Left*) and the neuron shown in **A** (*Right*). **D**: Statistical evaluation of G5-NH_2_ effects on astroglial Ca^2+^ enhancements: the number of cells showing Ca^2+^ enhancements (*Left*) and the average number of transients in 1 min (frequency) (*Right*). Horizontal bars represent significant differences at p < 0.05 level.

To investigate whether interaction of G5-NH_2_ with the plasma membrane of neurons and astroglia affects their function, we monitored intracellular Ca^2+^ signals that sensitively reflect the activity of both cell types. Astroglial Ca^2+^ signals were monitored in the astroglia-rich *stratum radiatum* area in the hippocampal CA3 region after bulk loading of the rat hippocampal slice with the Ca^2+^ sensitive fluorescent dye Fluo-4 [10,24] (Figure 2A-C, n = 7 slices). Astroglial localization of the dye was confirmed by colocalization with the astroglia-specific SR101 marker (Figure 2A, *Left*). Morphologically identified neurons in the CA3 pyramidal layer were directly filled with Fluo-4 from a patch pipette in order to visualize fine dendritic processes in addition to the cell body (Figure 2A, *Right,* n = 2 *cells*).

Application of G5-NH_2_ evoked Ca^2+^ enhancements in hippocampal astroglial cells (Figure 2B-D) suggesting increased astroglial activity. Ca^2+^ enhancements started almost immediately after G5-NH_2_ application (Figure 2C). To quantify the effect of G5-NH_2_ on the astroglial Ca^2+^ dynamics we determined the average number of responding cells per slice and the average frequency of Ca^2+^ transients (calculated from the intervals measured between subsequent peaks). Both parameters increased significantly during the 30 minute application period (Figure 2D). Number of responding cells and frequency of transients fell back to the control level during the washout period (Figure 2D) indicating reversible effect of G5-NH_2_ on astrocytes. In some cells, however, intracellular Ca^2+^ level remained slightly elevated (Figure 2C, *Left*, red and orange lines). In contrast, intracellular Ca^2+^ enhancements in pyramidal neurons were characterized by quickly developing, lasting increase in dendritic processes and almost linear increase of intrasomal Ca^2+^ level after 0.1 mg/ml G5-NH_2_ application (Figure 2C, *Right*).

### Distinguishable Ψ_MITO_ depolarization in neurons and astroglial cells

The observed transient or lasting enhancements of intracellular Ca^2+^ level after dendrimer application may result in impaired oxidative metabolism through Ca^2+^ influx-induced depolarization of Ψ_MITO_[13,14,16]. Mitochondrial cell death pathways have been suggested to contribute to the cytotoxic character of cationic PAMAM dendrimers in human lung cells [25]. In the brain, the neuronal activity and the mitochondrial function are highly correlated [15]. In addition, neuronal function and survival are very sensitive to mitochondrial dysfunction which can be monitored by measurement of the mitochondrial membrane potential [13,14,26]. To further explore this issue, we studied Ψ_MITO_ changes in astroglia and neurons using the Ψ_MITO_ sensitive dye rhodamine-123 [14].

**Figure 3:**
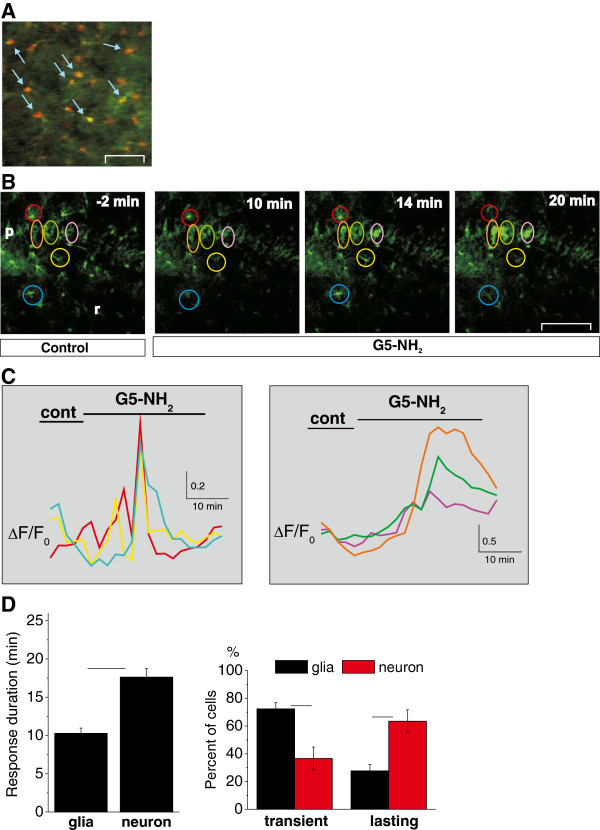
**G5-NH_2_ (0.1 mg/ml, 30 min) induces distinguishable mitochondrial membrane (Ψ_MITO_) depolarization in astroglial and neuronal cells as monitored with the fluorescent rhodamine-123 indicator. A**: Co-localization (yellow cells pointed by blue arrows) of astroglial cells stained with the astroglia-specific marker SR101 (red) with the Ψ_MITO_ depolarization indicator rhodamine-123 (green). **B**: Representative serial images showing Ψ_MITO_ depolarization in the circled cells in the CA3 *stratum pyramidale* (p) and CA3 *stratum radiatum* (r) areas of the rat hippocampal slice. Scale bar: 50 μm. **C**: Fluorescence-time plots of astroglial (*Left*) and neuronal (*Right*) cells circled in **B**. **D**: Statistical evaluation of astroglial vs. neuronal effects of G5-NH_2_ on Ψ_MITO_ depolarization dynamics: Ψ_MITO_ depolarization duration (*Left*) and percent of cells showing transient and lasting Ψ_MITO_ depolarization (*Right*). Asterisks represent significant differences at p < 0.05 level.

Application of G5-NH_2_ (0.1 mg/ml, 30 min) significantly increased the fluorescence of the Ψ_MITO_ sensitive dye rhodamine-123 in both pyramidal neurons and astroglia (Figure 3A-C, astroglial cells: n = 6 slices, neurons: n = 4 slices), indicating Ψ_MITO_ depolarization and impaired oxidative metabolism in both cell types. The dynamics of neuronal and astroglial response, however, showed distinctive features. Similarly to the Ca^2+^ responses, Ψ_MITO_ increase was found to be transient in most astroglial cells (Figure 3C and D) Responses were considered to be transient if the fluorescence intensity returned to ±20% of the baseline value within the application of G5-NH_2_. In contrast to astroglia, Ψ_MITO_ remained elevated in the majority of neurons until the end of the experiment (Figure 3C and D, lasting response), suggesting irreversible Ψ_MITO_ depolarization. In addition, the duration of the response was shorter (Figure 3D). Since astroglia is morphologically similar to interneurons, we completed colocalization experiments to identify astroglial cells. The observed colocalization of the astroglia specific fluorescent dye SR101 with Ψ_MITO_ depolarization monitored by the red fluorescent rhodamine-123 confirmed that, indeed, astroglial cells were probed (*c.f.* yellow color in the merged image Figure 3A).

**Figure 4:**
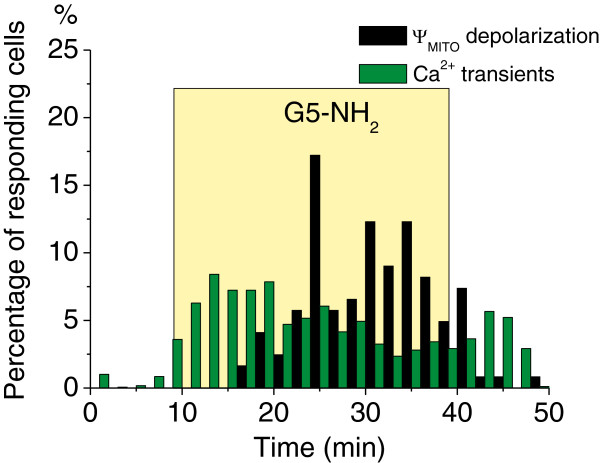
**Intracellular Ca^2+^ enhancements precede Ψ_MITO_ depolarization in astroglial cells during G5-NH_2_ (0.1 mg/ml) dendrimer application (yellow square) in the CA3 *stratum radiatum *area of the rat hippocampal slices.** Data represent the percentage of all responding astrocytes showing Ca^2+^ or Ψ_MITO_ transients at each time point.

To quantitatively compare the onset dynamics of Ca^2+^ enhancements and Ψ_MITO_ depolarization we determined the temporal distribution of Ca^2+^ transients (1783 Ca^2+^ transients were identified in 303 cells in 6 slices) and Ψ_MITO_ responses (122 Ψ_MITO_ peaks were measured in 144 cells in 7 slices) in all responding astroglialcells after G5-NH_2_ application (Figure 4). The appearance of intracellular Ca^2+^ transients was immediate and remarkably preceded Ψ_MITO_ depolarization (Figure 4) despite of the fact that the average number of responding astroglial cells per slice showing Ca^2+^ enhancements and Ψ_MITO_ depolarization was not significantly different (39 ± 7 vs. 24 ± 5, respectively; p = 0.117, one-way Anova). These data suggest a causal link between the two processes. It is to note that dynamics of intracellular Ca^2+^ transients and Ψ_MITO_ depolarization in pyramidal neurons has been shown to be coupled during seizure-like events [14].

### PAMAM dendrimer evokes astroglial Ψ_MITO_ depolarization directly and via neuron-astroglia interaction

**Figure 5:**
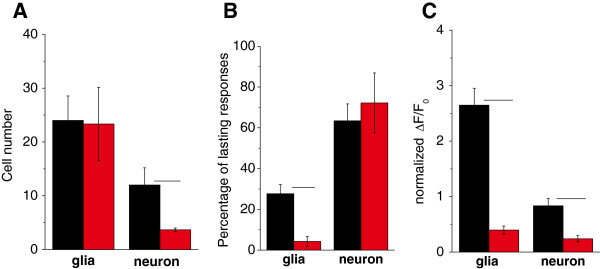
**Ψ_MITO_ depolarization after G5-NH_2_ (0.1 mg/ml, 30 min) application is comprised of neuronal activity-dependent and independent astroglial components.** Neuronal activity was blocked by TTX (1 μM), APV (100 μM), CNQX (10 μM) and picrotoxin (100 μM). Characteristic Ψ_MITO_ depolarization parameters, e.g. the number (**A**), length (**B**) and normalized fluorescence changes (**C**) of astroglial cells and neurons are shown. Fluorescence changes (ΔF/F_0_) were normalized to Ψ_MITO_ depolarization evoked by the mitochondrial inhibitor CCCP (10 μM). Asterisks represent significant differences at p < 0.05 level.

Since neuronal activation results in the release of major excitatory and inhibitory neurotransmitters Glu and γ-aminobutyric acid (GABA), respectively, and glutamat*ergic* activation can lead to Ψ_MITO_ changes [13,14]), we explored whether neuronal activation modifies astroglial responses. To examine whether G5-NH_2_ directly affects astroglial mitochondrial function or it is the consequence of the preceding neuronal depolarization, we measured G5-NH_2_ evoked Ψ_MITO_ depolarization in the presence of the following inhibitors: blocker of voltage-gated Na^+^ channels tetrodotoxin (TTX, 1 μM), antagonists of Glu receptors (N-methyl-D-aspartate type: DL-2-amino-5-phosphonopentanoic acid APV, 100 μM; AMPA/kainate type: 6-cyano-7-nitroquinoxaline-2,3-dion CNQX, 10 μM) and the GABA_A_ receptor antagonist picrotoxin (100 μM). In the presence of the antagonists, the number of astrocytes showing Ψ_MITO_ depolarization did not change, while the number of responding neurons significantly decreased (Figure 5A, astroglia n = 7 slices, neurons n = 3 slices). However, the blockade of neuronal activity decreased both the duration of the astroglial response (10.2 ± 0.7 min *vs.* 7.8 ± 0.8 min; p = 0.049, one-way Anova) and the percentage of lasting astroglial (but not the neuronal) Ψ_MITO_ depolarization (Figure 5B). The average intensity of ΔF/F_0_ changes in neurons and astrocytes were also significantly decreased (Figure 5C).

Neurons and astroglial cells are functionally interconnected within the brain. Increased neuronal activation could led to astroglial Ψ_MITO_ depolarization [13,14]. If astroglial Ψ_MITO_ depolarization found in our experiments is only the consequence of the G5-NH_2_-evoked neuronal activation then inhibition of neuronal activity should prevent Ψ_MITO_ depolarization in astroglia. Therefore the unchanged number of responding glial cells (Figure 5A) indicates that G5-NH_2_ directly evoked mitochondrial depolarization in astroglia, while the decreased duration (Figure 5B) and intensity (Figure 5C) in astroglial cells suggests that neuronal activation by G5-NH_2_ intensified the astroglial responses.

### Astrocytes are more resistant to PAMAM dendrimer neurotoxicity than neurons

**Figure 6:**
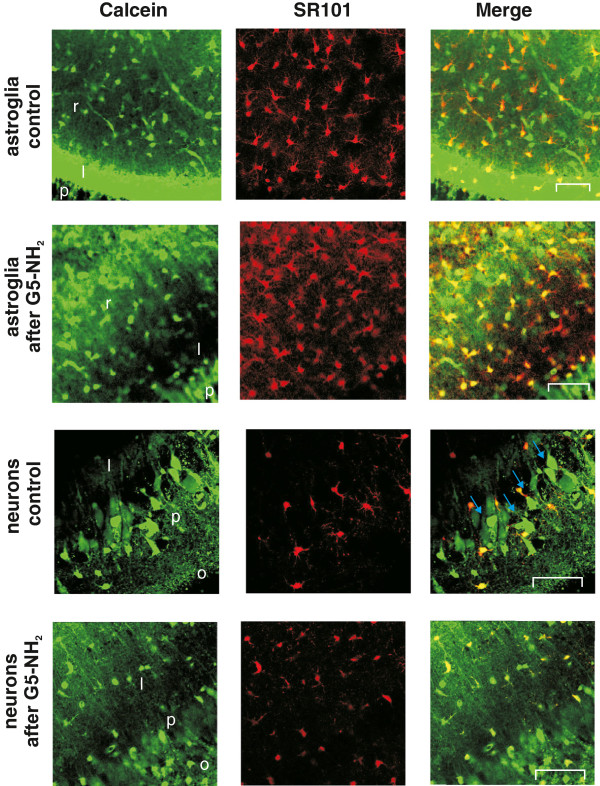
**Incubation of the rat hippocampal slice with 0.1 mg/ml G5-NH_2_ for 30 min differently affects the viability of astroglial cells and neurons.** Cells are stained with the fluorescent live-cell specific marker calcein-AM (green) and astroglia specific SR-101 (red). Merged images show viable astroglia (yellow) and viable neurons (green cells pointed by blue arrows). Scale bars: 100 μm. O: str. oriens; P: str. pyramidale; L: str. lucidum. Scale bars: 100 μm.

Lasting Ψ_MITO_ depolarization of neuronal and some astroglial cells might indicate irreversible disturbances of cellular metabolism [13-15,27]. Predominantly shorter astroglial responses, however, suggest that G5-NH_2_ application might be less harmful to astrocytes probably because astroglial Ψ_MITO_ can be recovered after several minutes of depolarization [26]. To assess the consequence of G5-NH_2_ induced Ψ_MITO_ depolarization we measured the viability of astrocytes and neurons by labeling the live cells with calcein after 30 min exposure to G5-NH_2_. The SR101 positive astroglial cells showed robust calcein fluorescence in the *stratum radiatum* after 30 min of G5-NH_2_ application indicating the presence of functional, viable astroglial cells [28] (Figure 6A *Top,* n = 3 slices), although viability of astrocytes in the *stratum lucidum* region may also be compromised. Contrary, in accordance with our previous observations [13], a large proportion of hippocampal pyramidal neurons lost their viability after 30 min application of G5-NH_2_ despite the survival of astrocytes in the same region (Figure 6*Middle* and *Bottom*). These findings are in accordance with the neuronal activity-dependent lasting Ψ_MITO_ (*c.f.* Figure 4) and plasma membrane [7] depolarization. Transient as opposed to lasting Ψ_MITO_ depolarization in astroglia and neurons, respectively, indicates that the neurotoxicity [7] of G5-NH_2_ may predominantly be restricted to neurons over astroglia.

## Conclusion

G5-NH_2_ activates both astrocytes and neurons in acute hippocampal slices as reflected by intracellular Ca^2+^ enhancement and Ψ_MITO_ depolarization. We showed that the interaction of PAMAM dendrimer with the plasmamembrane evokes Ψ_MITO_ depolarization most probably via the enhancement of intracellular Ca^2+^ level. Vast majority of astrocytes shows transient response and remains viable. In contrast, lasting activation of neurons by G5-NH_2_ provokes fatal consequences in accordance with the predominantly irreversible early depolarization of neurons [7]. Due to the connection between elevated Ca^2+^ signal and Ψ_MITO_ depolarization, as well as formation of reactive oxygen species [11,13,16,27], we can also infer the early disturbance of oxidative metabolism as the primary cause of PAMAM dendrimer evoked neuronal toxicity.

## Methods

### Chemicals

PAMAM dendrimer (G5-NH_2_) was purchased from Dendritech Inc. (Dendritech.com, USA). All other chemicals were obtained from Sigma-Aldrich unless otherwise stated.

### Slice preparation

Animal experiments were carried out in accordance with the European Communities Council Directive of 24 November 1986 (86/609/EEC) and the Hungarian Animal Act, 1998 and associated local guidelines. Transverse 400 μm thick hippocampal slices of juvenile (10–16 days old) male Wistar rats (Toxicoop, Budapest, Hungary) were prepared as described elsewhere [7]. Slices were submerged and perfused at 2 ml/min by artificial cerebrospinal fluid (ACSF, composition in mM: 129 NaCl, 10 glucose, 3 KCl, 1.25 NaH_2_PO_4_, 1.8 MgSO_4_, 2 CaCl_2_ and 21 NaHCO_3_), saturated with carbogen (5% CO_2_ + 95% O_2_), pH 7.4.

### Imaging

In order to monitor changes in intracellular Ca^2+^, rat brain hippocampal slices were incubated with 5 μM Fluo-4 AM in ACSF for one hour at 35°C in the dark under humidified carbogen atmosphere after preincubation in 2% pluronic acid containing ACSF for 2 minutes [10]. To allow the cleavage of the AM ester group of Fluo-4, slices were transferred to dye-free ACSF at least 30 minutes before the start of the experiment.

In order to monitor changes in Ψ_MITO_ rat brain hippocampal slices were loaded with the fluorescent Ψ_MITO_ indicator rhodamine-123 (15 μg/ml in ACSF) for 20 minutes at 25°C [14]. To identify astrocytes slices were loaded with sulforodamine-101 immediately after slicing (1 μM, 20 minutes, 35°C, [29]) before rhodamine-123 loading.

Dye-loaded slices were placed into the observation chamber and superfused with ACSF and G5-NH_2_ (0.1 mg/ml in ACSF). Change in Fluo-4 and rhodamine-123 fluorescence (λ_ex_ = 488 nm, λ_em_ = 510–530 nm) and SR101 (λ_ex_ = 543 nm, λ_em_ = 570–660 nm) was imaged by a confocal laser scanning microscope (FluoView300, Olympus, Hungary) by 2 min or 10 sec image intervals for rhodamine-123 and Fluo-4 labeling, respectively. Average values of responding astroglial cells per slice showing Ψ_MITO_ depolarization and Ca^2+^ enhancements were 24 ± 5 and 39 ± 7 respectively. Control images were taken for 8 minutes of ACSF perfusion followed by 30 minutes application of 0.1 mg/ml G5-NH_2_ and a 10 minute washout period (in Fluo-4 experiments). In Ψ_MITO_ experiments carbonyl cyanide 3-chlorophenylhydrazone (CCCP, 10 μM in 0.1% DMSO) was applied at the end of the measurement to determine rhodamine-123 intensity corresponding to total Ψ_MITO_ depolarization.Under control conditions, rhodamine-123 enters mitochondria and, due to self-quenching, the overall fluorescence is low. When the mitochondria depolarize, dye leaves the mitochondria resulting in fluorescence enhancement [15,29].

Astroglial cell viability was measured using Calcein-AM fluorescent dye (λ_ex_ = 488 nm, λ_em_ = 510–530 nm). The intracellular esterase activity could be used as a probe of viability and plasma membrane competence and as an indicator of the cellular functionality [28]. Calcein-AM is a membrane-permeable non-fluorescent molecule that enters intact living cells, then it is cleaved by endogenous esterases to produce the highly fluorescent, membrane impermeable molecule, calcein.

### Data evaluation

Images recorded by the FluoView300 software were processed using the free ImageJ 1.41 image analysis software (http://rsbweb.nih.gov/ij/). Matlab 6.1 was used to evaluate fluorescence changes and the number of responding cells and frequency of fluorescent transients. To avoid differences between slices G5-NH_2_ evoked changes in fluorescence intensity (ΔF/F_0_) were normalized to the average response of the cells to 10 μM CCCP, a well known mitochondrial inhibitor applied at the end of the experiments (ΔF/F_0_ after CCCP application was 2.2 ± 0.4 for astroglial cells and 1.5 ± 0.16 for neurons). Data presented are mean ± S.E.M. Statistical analysis was performed using one-way Anova (OriginLab Co., Northampton, UK) and p < 0.05 was considered statistically significant.

### Synthesis of fluorescently labeled G5-NH_2_

Rhodamine Green was covalently bound to G5-NH_2_ by reacting aqueous solution of G5-NH_2_ (1400 μl, 1.97 μmol, 4.05 w/w%) with Rhodamine Green carboxylic acid succinimidyl ester hydrochloride mixed isomers (5(6)-CR 110, SE; Molecular Probes, Eugene, OR, USA) (1 mg, 1.97 μmol) dissolved in *N,N*-dimethylformamide (100 μl) in 0.1 M NaHCO_3_ buffer (1.4 ml, pH 8.5) at room temperature for 2 h in dark. The unreacted dye was removed from the solution by ultrafiltration (3 000 MWCO) in Amicon Ultracel – 3K centrifugal filter units. Amine reactive form of the Rhodamine Green dye was coupled covalently to the PAMAM dendrimer forming amide bonds. The unreacted dye was then removed by ultrafiltration and the conjugate containing no dye was used throughout the experiments. The conjugate is hydrolitically stable under physiological conditions therefore the localization of the dendrimer can be interpreted by the detected fluorescence.

## Abbreviations

G5-NH2: PAMAM generation 5 dendrimer; ΨMITO: mitochondrial membrane potential; SR101: Sulforhodamine-101; TTX: Tetrodotoxin; APV: DL-2-Amino-5-phosphonopentanoic acid; CNQX: 6-cyano-7-nitroquinoxaline-2,3-dion; CCCP: Carbonyl cyanide 3-chlorophenylhydrazone.

## Competing interests

The authors declare that they have no competing interests.

## Authors’ contributions

Conceived and designed the experiments: GNy, LH, JK. Performed the experiments: GNy, IP. Analyzed the data: GNy. Synthetized and filtrated the fluorescently labeled dendrimer: IJ. and JV. Wrote the paper: GNy, LH, JK. All authors read and approved the final manuscript.
